# Inhibition of Pirfenidone on TGF-beta2 Induced Proliferation, Migration and Epithlial-Mesenchymal Transition of Human Lens Epithelial Cells Line SRA01/04

**DOI:** 10.1371/journal.pone.0056837

**Published:** 2013-02-21

**Authors:** Yangfan Yang, Yiming Ye, Xianchai Lin, Kaili Wu, Minbin Yu

**Affiliations:** State Key Laboratory of Ophthalmology, Zhongshan Ophthalmic Center, Sun Yat-sen University, Guangzhou, China; Astar-Neuroscience Research Partnership (NRP) and Institute of Medical Biology (IMB), Singapore

## Abstract

**Background:**

Posterior capsular opacification (PCO) is a common complication of cataract surgery. Transforming growth factor-β2 (TGF-β2) plays important roles in the development of PCO. The existing pharmacological treatments are not satisfactory and can have toxic side effects.

**Methodologies/Principal Findings:**

We evaluated the effect of pirfenidone on proliferation, migration and epithlial-mesenchymal transition of human lens epithelial cell line SRA01/04 (HLECs) in vitro. After treatment with 0, 0.25, and 0.5 mg/ml pirfenidone, cell proliferation was measured by MTT assay. Cell viability was determined by trypan blue exclusion assay and measurement of lactate dehydrogenase (LDH) activity released from the damaged cells. And cell migration was measured by scratch assay in the absence or presence of transforming growth factor-β2 (TGF-β2). The expressions of TGF-β2 and SMADs were evaluated with real-time RT-PCR, western blot, and immunofluorescence analyses. The mesenchymal phenotypic marker fibronectin (FN) was demonstrated by Immunocytofluorescence analyses. The cells had high viability, which did not vary across different concentrations of pirfenidone (0 [control] 0.3, 0.5 or 1.0 mg/ml) after 24 hours. Pirfenidone (0∼0.5 mg/ml) had no significant cytotoxicity effect on SRA01/04 by LDH assay. Pirfenidone significantly inhibited the proliferation and TGF-β2-induced cell migration and the effects were dose-dependent, and inhibited TGF-β2-induced fibroblastic phenotypes and TGF-β2-induced expression of FN in SRA01/04 cells. The cells showed dose-dependent decreases in mRNA and protein levels of TGF-β2 and SMADs. Pirfenidone also depressed the TGF-β2-induced expression of SMADs and blocked the nuclear translocation of SMADs in cells.

**Conclusion:**

Pirfenidone inhibits TGF-β2-induced proliferation, migration and epithlial-mesenchymal transition of human lens epithelial cells line SRA01/04 at nontoxic concentrations. This effect may be achieved by down regulation of TGF-β/SAMD signaling in SRA01/04 cells.

## Introduction

Posterior capsular opacification (PCO) is the most frequent complication of cataract surgery and mainly caused by fibrosis on lens capsular bag and/or posterior capsule [1]. Previous studies have shown that many growth factors, especially transforming growth factor-β2 (TGF-β2), play important roles in the development of PCO, which may trigger a series of events (including cell proliferation, differentiation, migration and capsular fibrosis) during wound healing process [2]–[5]. Blockage of TGF-β2 signaling may represent a means to prevent the development of PCO.

Many methods have previously been developed to prevent the development of such fibrosis. These include topical administration of anti-inflammation medications (e.g., dexamethasone, heparin, diclofenac) [6]–[8] or anti-metabolic agents (e.g., 5-fluorouracil, mitomycin) [9], [10], improved design of intraocular lens (IOL) [11], [12], new IOL materials, and modified surgical procedures (e.g., bag-in-the-lens IOL implantation, optic buttonholing) [13]–[15]. However, none of the pharmacological therapies is effective and safe enough for the prevention of PCO, and some of them are toxic [16], [17]. New IOLs and modified surgical methods have led to a significant reduction of PCO in some, but not all patients^16^, and they are costly and may not be readily available in low-income countries.

Pirfenidone (PFD) is a novel broad-spectrum anti-inflammatory and anti-fibrosis agent, and it has been widely used as an oral formulation for systemic treatment of idiopathic pulmonary fibrosis (IPF) [18]. Our research team has previously shown that PFD can prevent the proliferation and migration of cultured human Tenon capsule fibroblasts (HTFs) and reduce fibrosis in a rabbit model of glaucoma filtration surgery, and pharmacokinetic analysis showed that PFD can be used as a topical eye drop. Toxicity assessments showed that PFD did not damage the rabbit eyes [19]–[21].

In the current study, we hypothesized that PFD can suppress the proliferation and migration of human lens epithelial cells (HLECs). We investigated the effects of PFD on proliferation and migration of HLECs. Considering the important effect of TGF-β2 signaling pathway, we examined the roles of PFD on mRNA or protein expression TGF-β2 and SMADs and the nuclear translocation of SMADs.

## Materials and Methods

### Cell Culture

The SV40 T-antigen-transformed human lens epithelial cell line [22], SRA01/04, was obtained from Cancer Institute of Chinese Academy of Medical Sciences. Cells were cultured in modified Eagle’s medium (α-MEM; Hyclone, Logan, UT) supplemented with 10% fetal bovine serum (FBS) and 1% non-essential amino-acid (NEAA) at 37°C in a humidified atmosphere containing 5% CO2. Culture plates were coated for 30 min at 37°C with gelatin solution before cell inoculation.

### MTT Assay

HLECs were seeded in 96-well plates (1.0×10^4^ cells/well) for 24 hours in α-MEM/10% FBS/1%NEAA, and were cultured in stationary tubes in serum-free medium for 24 hours. And then the culture medium was removed and cells were bathed in α-MEM with 10% FBS and 1% NEAA supplemented with 0, 0.01, 0.1, 0.2, 0.3, 0.5, or 1 mg/ml PFD (Sigma-Aldrich, St. Louis, MO) for 0, 4, 12, 24, 48, or 72 hours. After incubation with 180 µL α-MEM and 20 µL of 5 mg/ml 3-[4, 5-dimethylthiazol-2-yl] -2, 5-diphenyl tetrazolium bromide (MTT, Amresco, Solon, OH) for 4 hours at 37°C, the MTT solution was discarded. The Formosan precipitates were dissolved in 180 µL DMSO (Amresco, Solon, OH) by agitating the dishes for 10 minutes at 200 rpm on an orbital shaker. The absorbance at 490 nm in each well was read with a micro plate reader (Bio-Tek, Vermont, U.S.A). We further examined the effects of PFD by refining the concentrations at 0.2, 0.25, 0.3, 0.4, 0.5 and 0.6 mg/ml using the MTT assay.

### Cell Viability Assay

Cell viability was evaluated by both trypan blue exclusion method and measurement of lactate dehydrogenase (LDH) activity released from the cytosol of damaged cells. In trypan blue exclusion test, HLECs were treated with PFD at the concentrations of 0, 0.3, 0.5 or 1 mg/ml. Cell viability was measured within 24 hours. After dye with trypan blue, stained (dead) and unstained (viable) cells were counted with a hematocytometer. The percentage of living cell was calculated according to the following formula: % cell viability = (viable cell count/total cell count)×100. In LDH assay, LDH activity release from the demaged cells was measureed after treatment with 0.25 mg/ml and 0.5 mg/ml PFD. The cultrue medium was collected and a Cytotoxicity Detection Kit (Roche, Penzberg, Germany) was used for the assay. The percentage of cell-mediated lysis was expressed asaccording to the following formula: % cytotoxicity = (exp. value – low control)/(high control – low control).

### Cell Migration Assay

We use scarification test to measure the migration ability of the cells. First, the HLECs were grown to a confluent monolayer and then placed in serum-free medium for 24 hours. After the medium was discarded, we make a straight line scratch across the cell layer with a p20 pipette tip and then rinsed with Phosphate buffered saline (PBS) to remove the suspended cells and treated with 0, 0.25 or 0.5 mg/ml PFD in the absence or presence of 12.5 ng/ml TGF-β2 (R&D System, Minneapolis, MN) for 24 hours. The scratch gap was recorded and photographed after 24 hours under a light microscope and analyzed with an image processing software (Photoshop 7.0; Adobe, San Jose, CA).

### Real-time Reverse Transcription-polymerase Chain Reaction

HLECs were treated with PFD at the concentrations of 0, 0.25, or 0.5 mg/ml for 24 hours, and then the RNA was isolated from cultured cells with TRIzol reagent (Invitrogen, Carlsbad, CA). Reverse transcription - polymerase chain reaction (RT-PCR) was carried out using the one-step reverse transcription-polymerase chain reaction system (TakaLa, Dalian, China). The primer pairs which we used were listed in **Table 1**.

**Table 1:** Human Primer Sequences Used for Realtime RT-PCR.

Gene	Sense Primer	Antisense Primer	Probe (bp)
TGFβ2	GAGGGATCTAGGGTGGAAATGG	AGGACCCTGCTGTGCTGAGT	104
SMAD3	TCGAGCCCCAGAGCAATATT	CGTCCATGCTGTGGTTCATC	97
SMAD4	ACATTGGATGGGAGGCTTCA	GATCAGGCCACCTCCAGAGA	95
β-actin	GCATGGGTCAGAAGGATTCCT	TCGTCCCAGTTGGTGACGAT	106

### Western Blot Assay

HLECs were treated with 0, 0.25, or 0.5 mg/ml PFD in the absence or presence of 12.5 ng/ml TGF-β2 for 24 hours. The cells were dispersed in mammalian protein extraction reagent (M-PER; Bioteke, Beijing, China). Final protein concentrations were determined with the BCA protein assay kit (Biocolor, Shanghai, China) according to the manufacturer’s instructions. Prepared samples were heated to 100°C for 5 minutes; same amount of protein was added to a well of an 8–12% acrylamide gel according to molecular weight of targeted proteins and resolved by SDS-PAGE. The separate proteins were then transferred to a Polyvinylidene Fluoride membrane (PVDF membrane; Millipore, Billerica, MA). Nonspecific binding was blocked by incubation with 5% nonfat milk for 2 hours before overnight incubation with primary antibodies (Abcam, Cambridge, UK) (1∶1000 anti-TGF-β2, 1∶500 anti-SMADs) at 4°C. After it was washed, the membrane was incubated with 1∶5000 dilution of secondary antibody conjugated with horseradish peroxidase (Jetway Biotech, Guangzhou, China) in PBS-Tween. Blots were developed by chemiluminescence, to produce a signal that was captured on Chemi-Smart imaging systems (Vilber Lourmat, Marne-la-Vallée, France) according to the manufacturer’s instructions, and were then scanned for densitometric analysis (ImageQuant software, version 5.2; Molecular Dynamics, Sunnyvale, CA).

### Immunofluoresence Assay

HLECs were treated with 0, 0.25, or 0.5 mg/ml PFD in the absence or presence of 12.5 ng/ml TGF-β2 on coverslips for 24 hours. They were fixed with pure methanol for 10 minutes at −20°C followed by several rinses in PBS. Nonspecific binding sites were blocked for 30 minutes in PBS with 5% goat serum (Ruite, Guangzhou, China). The cells were incubated overnight at 4°C with primary antibodies (Abcam, Cambridge, UK) at 1∶200, and then incubated with secondary antibodies conjugated with Alexa Fluor 488 or Alexa Fluor 594 (Invitrogen, Carlsbad, CA) for 30 minutes in dark at room temperature, followed by incubation with 4′,6-Diamidino-2-phenylindole dihydrochloride (DAPI, Sigma-Aldrich, St. Louis, MO) for 5 minutes. The cells were subsequently scanned with a confocal microscope (Zeiss, LSM 510 META).

### Statistical Analysis

All results are expressed as the mean ± SD. Changes in outcome across the various time points in MTT assay were assessed using repeated measure analysis of variance. One-way analysis of variance (ANOVA), the test of homogeneity of variances, and a post hoc test (Bonferroni test) were used to determine significant differences between groups. A *P*<0.05 was considered statistically significant.

## Results

### Inhibitory Effects of PFD on Cell Proliferation of SRA01/04 Cells

**Figure 1:**
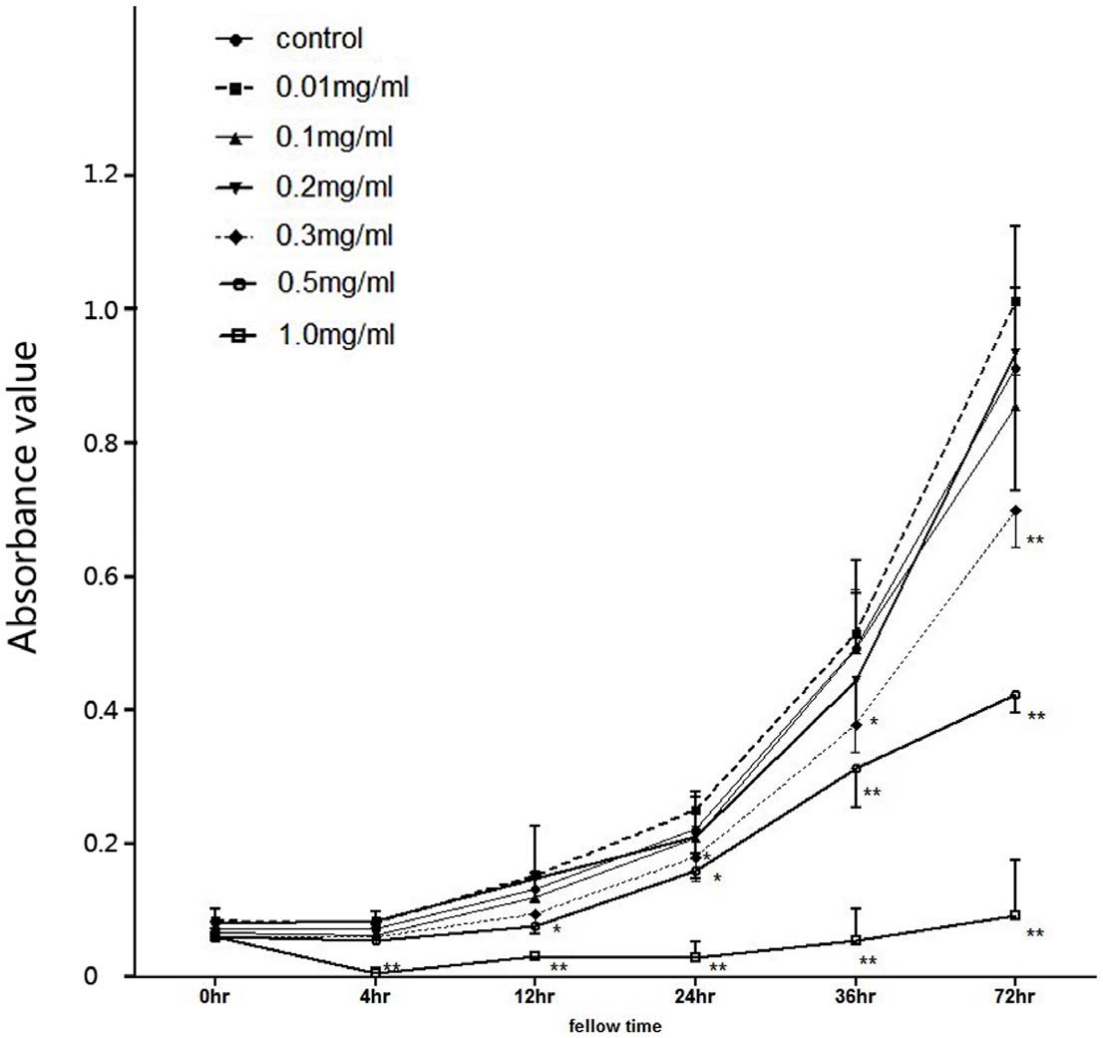
Inhibitory effect of pirfenidone on proliferation of SRA01/04 cells as measured by MTT assay. Cells with 10% FBS were treated with 0, 0.01, 0.1, 0.2, 0.3, 0.5, or 1.0 mg/mL pirfenidone for 0, 4, 12, 24, 48, or 72 hours. Data are derived from the mean ± SD of triplicate results in three independent experiments. *P<0.05, and **P<0.001 from comparisons between cells with treatment and control cells with 10% FBS, at different time points.

PFD showed its inhibitory effects on the proliferation of HLECs (**Figure 1**). Cell proliferation was attenuated in the 0.3 mg/ml group after 24 hours compared with the control group (*P = *0.044). The effect was more apparent in the 0.5 mg/ml group at 24, 48, and 72 hours (*P*<0.05). The proliferation was almost completely inhibited with 1 mg/ml PFD at all the time-points (*P*<0.01). The assays were repeated by refining the concentration at 0.2, 0.25, 0.3, 0.4 0.5 and 0.6 mg/ml in three independent experiments. After 24 hours treatment, the inhibition started at 0.25 mg/ml and reached a maximum effect at 0.5 mg/ml. The fifty percent inhibiting concentration (IC50) of PFD on HLECs proliferation was 0.47. According to repeated measure analysis of variance, there was significant interaction between the PFD concentration and duration (*P*<0.01). Given these results, the following experiments were carried out with the use of 0.25 and 0.5 mg/ml PFD with 24 hours of observation.

### Cell Viability of SRA01/04 Cells after Treated with PFD

**Figure 2:**
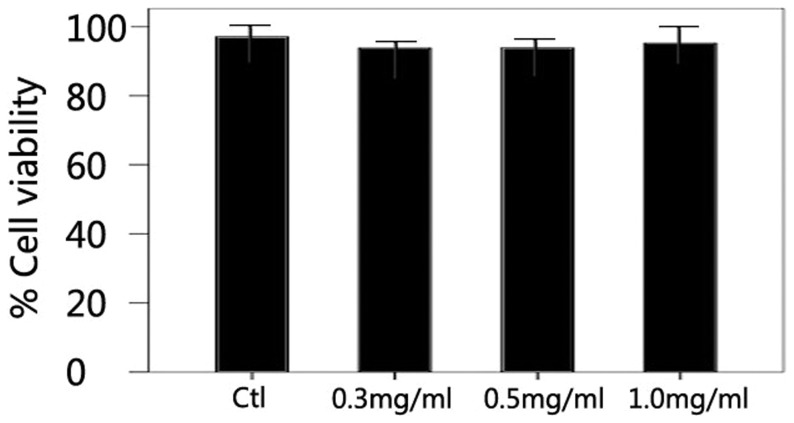
Cell viability of SRA01/04 cells after treated with pirfenidone for 24 hours in a trypan blue exclusion test. After 24 hours’ treatment with PFD (0, 0.3, 0.5, and 1 mg/ml), the percentages of living cells were more than 90% in all groups. No statistically significant difference was found between PFD treated and control groups (*P*>0.05). Data are the mean ± SD of triplicates from an experiment that was repeated with similar results.

In trypan blue exclusion test, after 24 hours’ treatment with PFD, the percentages of living cells were 97.06±4.3%, 93.75±1.6%, 93.85±2.9%, and 95.16±4.8% for the control, 0.3, 0.5, and 1 mg/ml PFD groups, respectively (**Figure 2**). There was no statistically significant difference between the groups (P>0.05).

**Figure 3:**
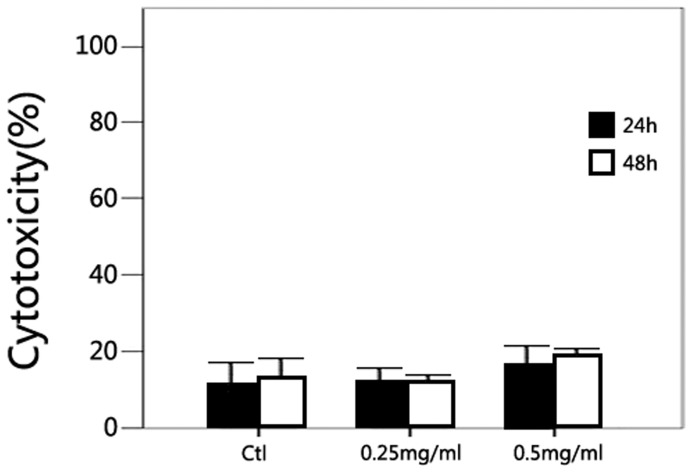
Cytotoxicity of pirfenidone in a LDH assay. After 24 or 48 hours’ treatment with PFD (0, 0.25, and 0.5 mg/ml), no statistically significant difference was found between the percentage of cell-mediated lysis in PFD treated groups and control group (*P*>0.05). Data are the mean ± SD of triplicates from an experiment that was repeated with similar results.

In LDH assay, after 24 hours’ treatment with PFD, the percentages of cell-mediated lysis were 13.22±5.3%, 14.42±3.9%, and 18.18±3.3% for the control, 0.25 and 0.5 mg/ml PFD groups, and the percentages of cell-mediated lysis after 48 hours’ were 16.17±3.1%, 14.51±1.9%, and 20.37±1.6% for the control, 0.25 and 0.5 mg/ml PFD groups. PFD had no significant cytotoxicity effect on SRA01/04 cells (P>0.05) (**Figure 3**).

### PFD Inhibited TGF-β2-induced Fibroblastic Phenotypes and Epithlial-mesenchymal Transition in SRA01/04 Cells

**Figure 4:**
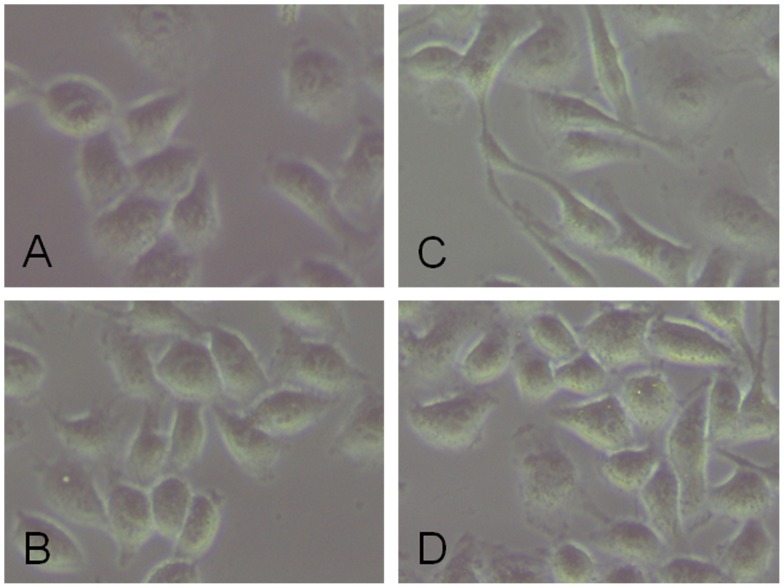
Inhibitory effect of pirfenidone on TGF-β2-induced fibroblastic phenotypes in SRA01/04 cells. There was no apparent morphological change in SRA01/04 cells after 24 hours’ treatment with 0.5 mg/ml PFD only (B) compared with control cells(A). In the presence of 12.5 ng/ml TGF-β2, the TGF-β2-induced morphological changes in SRA01/04 cells (C) were detected, including elongated and spindle-like shapes, which were noticeably suppressed by co-treatment with 0.5 mg/ml PFD (D).

After 24 hours’ treatment with 12.5 ng/ml TGF-β2, the TGF-β2-induced morphological changes in SRA01/04 cells were detected. The cells were elongated and began to be spindle-like shapes. The morphological changes induced by TGF-β2 were noticeably suppressed by co-treatment with 0.5 mg/ml PFD for 24 hours. There was no apparent morphological change in SRA01/04 cells after 24 hours’ treatment with 0.5 mg/ml PFD only. (**Figure 4**).

**Figure 5:**
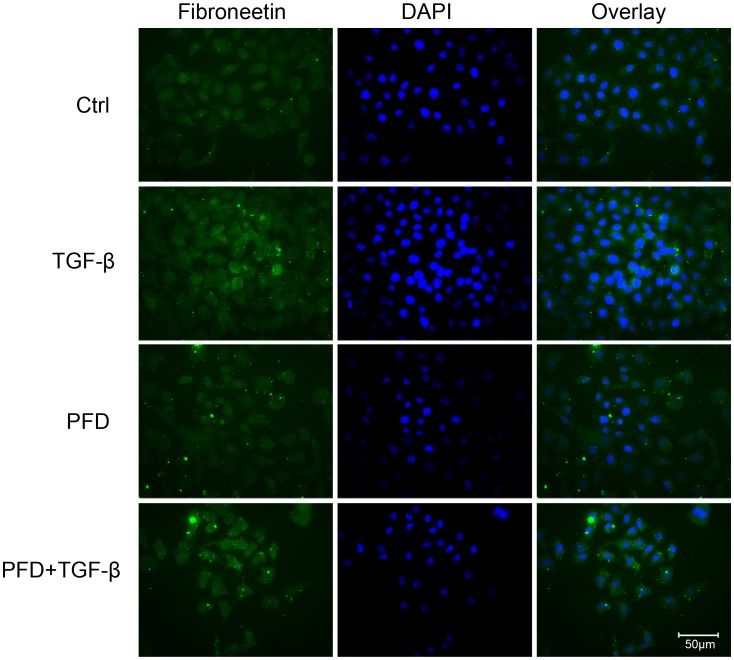
Inhibitory effect of pirfenidone on TGF-β2-induced epithlial-mesenchymal transition in SRA01/04 cells. Compared with control cells (A), immunocytofluorescence demonstrated that the TGF-beta2 significantly up-regulated the mesenchymal phenotypic marker fibronectin (FN, green) in SRA01/04 cells (C). Either in the absence (B) or presence (D) of TGF-beta2, FN was significantly down-regulated after 24 hours’ treatment with 0.5 mg/ml PFD. The nuclei stained by DAPI (blue). Magnification, ×400.

Immunocytofluorescence demonstrated that the TGF-beta2 significantly up-regulated the mesenchymal phenotypic marker fibronectin (FN) in SRA01/04 cells compared with control cells. And FN was significantly down-regulated after 24 hours’ treatment with 0.5 mg/ml PFD in the absence or presence of 12.5 ng/ml TGF-beta2. (**Figure 5**).

### Inhibitory Effect of PFD on the TGF-β2-induced Migration of SRA01/04 Cells

**Figure 6:**
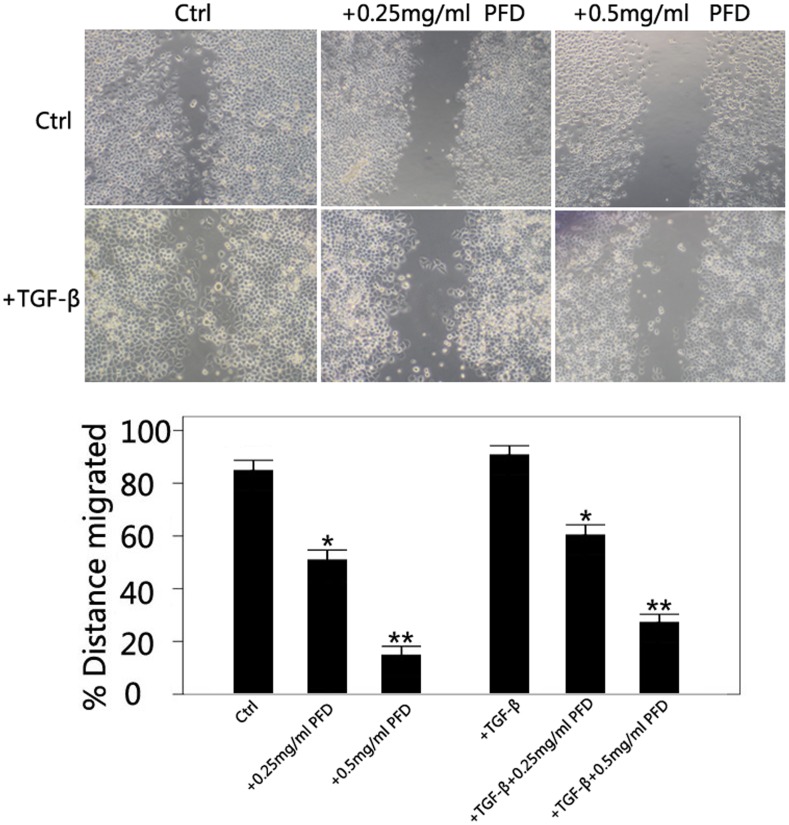
Inhibitory effect of pirfenidone on TGF-β2-induced migration of SRA01/04 cells. Light microscope images show decreased migratory ability of cells at 24 hours, after scratches were applied to the cells with 0, 0.25, or 0.5 mg/mL pirfenidone in the absence or presence of TGF-β2. *P<0.05 and **P<0.01 versus the corresponding value for control cells. Data in each bar are the mean ± SD of cells that migrated through the membrane in three separate experiments. Magnification, ×40.

The migration of HLEC was significantly reduced after of the treatment of PFD (0.25 and 0.5 mg/ml) for 24 hours (**Figure 6**) in the absence or presence of 12.5 ng/ml TGF-β2. In the absence of TGF-β2, the migration rates were 84.9%±3.1%, 51.0%±3.4%, and 14.9%±3.4% for the control, 0.25 and 0.5 mg/ml groups, respectively. In the TGF-β2-treated groups, the migration rates were 91.3%±2.8%, 60.9%±3.2%, and 26.1%±2.7% for the control, 0.25 and 0.5 mg/ml groups, respectively (*P*<0.05 between groups).

### Effect of PFD on Expression of TGF-β2 and SMADs mRNA and Protein in SRA01/04 Cells

**Figure 7:**
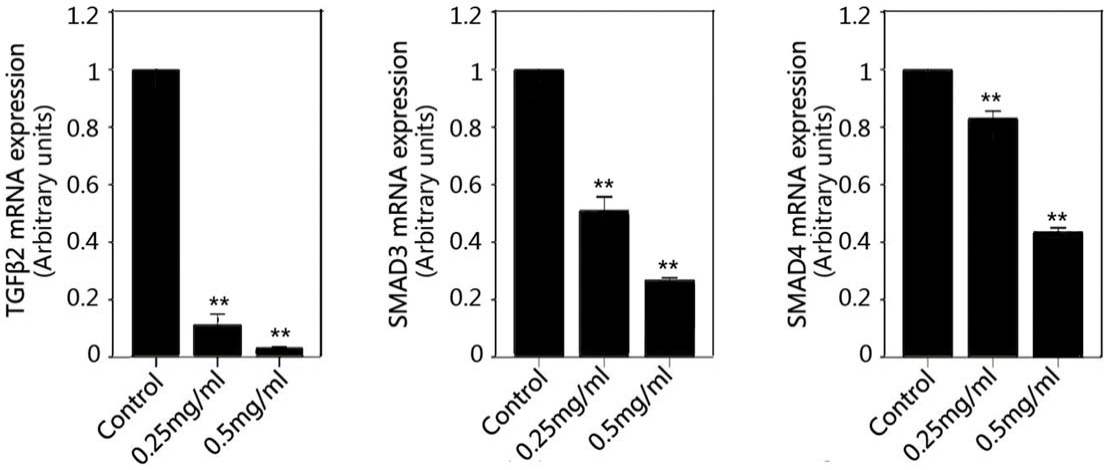
Inhibitory effect of pirfenidone on expression of TGFβ2 and SMADs mRNA in SRA01/04 cells. Real-time RT-PCR showed that pirfenidone reduced the levels of TGFβ2, SMAD3, and SMAD4 when compared with control cells. **P<0.01 versus the corresponding value for control cells. Data are the mean ± SD of triplicates from an experiment that was repeated with similar results.

**Figure 8:**
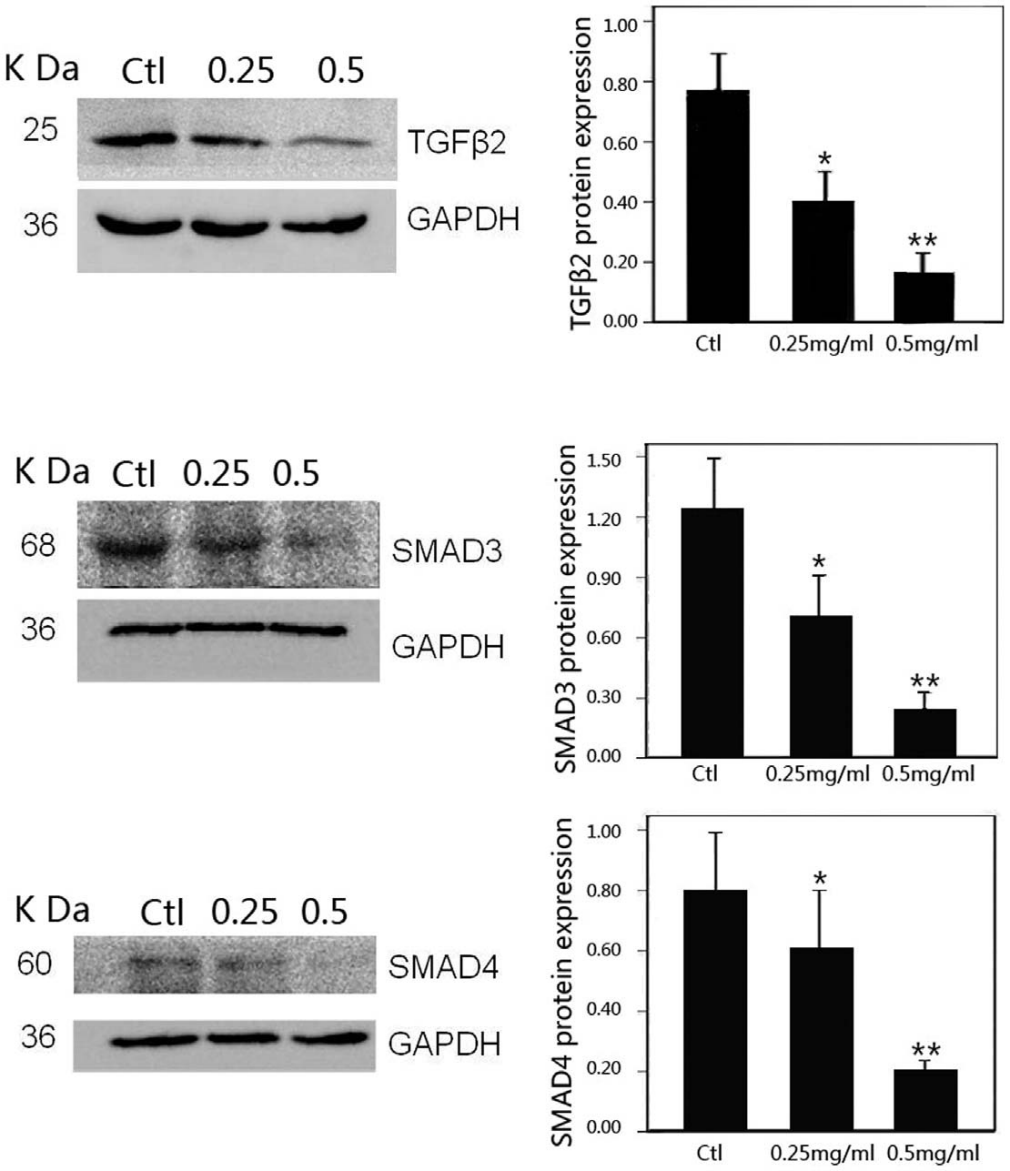
Inhibitory effect of pirfenidone on expression of TGFβ2 and SMADs protein in SRA01/04 cells. Western blot showed that pirfenidone reduced the protein levels of TGFβ2, SMAD3 and SMAD4 when compared with control cells. *P<0.05 and **P<0.01 versus the corresponding value for control cells. Data are mean ± SD of results from three independent cultures.

**Figure 9:**
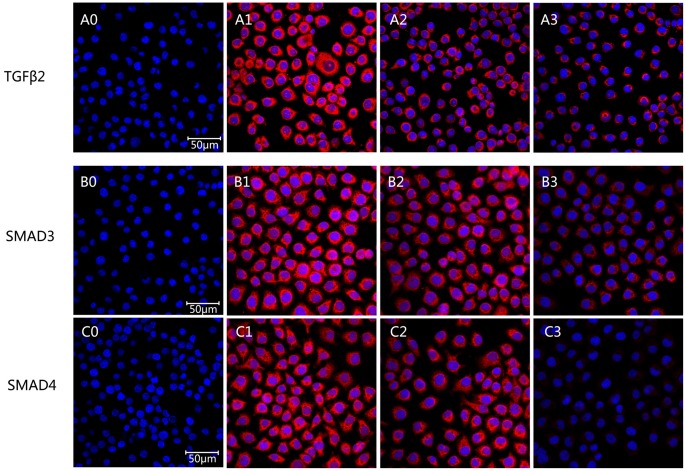
Expression of TGFβ2 and SMADs protein in SRA01/04 cells after treated with pirfenidone by immunocytochemical detection under confocal microscopy. TGFβ2 (A), SMAD3 (B), and SMAD4 (C) in the cytoplasm (red) and nuclei (blue) of HLECs were stained by immunocytochemistry after treatment with 0.25 (A2, B2, C2) and 0.5 mg/mL (A3, B3, C3) pirfenidone, respectively. (A0, B0, C0) Corresponding negative controls. Magnification, ×400.

A significant down-regulation of TGF-β2, SMAD3 and SMAD4 mRNA in HLECs was shown in the PFD treated groups (*P*<0.01) compared with the control (**Figure 7**). The expressions of TGF-β2, SMAD3 and SMAD4 protein were inhibited significantly at the concentration of 0.25 (*P*<0.05) and 0.5 mg/ml (*P*<0.01) compared with the control by western blot analyses (**Figure 8**). The effect was dose-dependent. We scanned the expression of TGF-β2, SMAD3 and SMAD4 protein in HLECs also by immunofluorescence assay under confocal microscopy (**Figure 9**). These proteins could be detected expressing inside the cells and almost in the cytoplasm according to the immunofluorescence assay. After treated with 0.25 and 0.5 mg/ml PFD for 24 hours, TGF-β2, SMAD3 and SMAD4 protein stained by immunocytochemistry in HLECs were very faint under confocal microscopy.

### Inhibitory Effect of PFD on TGF-β2-induced Expression of SMADs Protein

**Figure 10:**
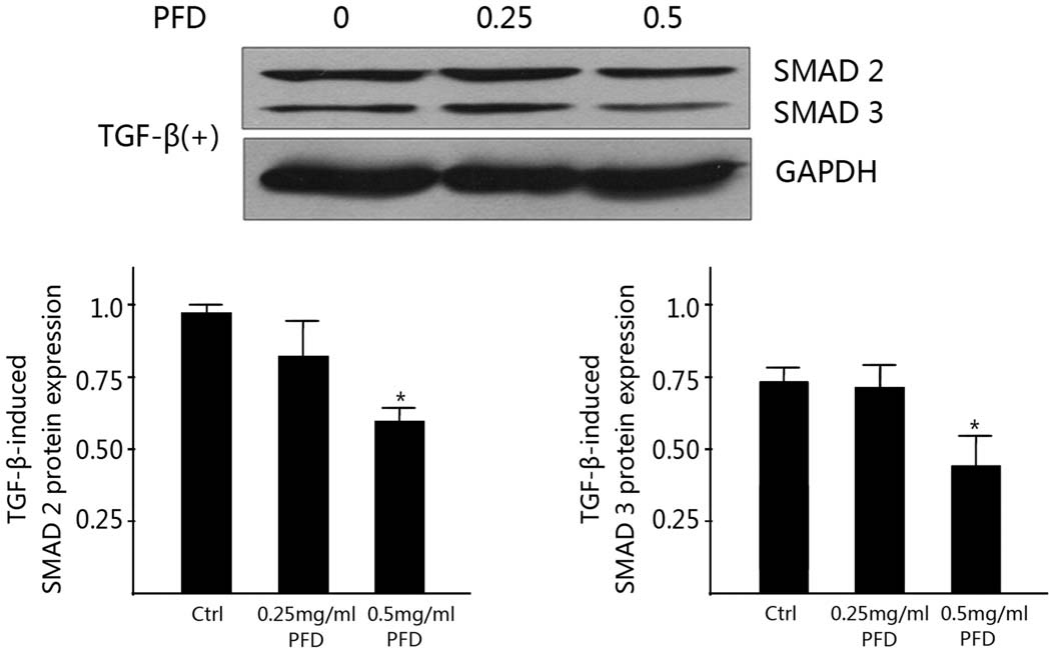
Inhibitory Effect of PFD on TGF-β2-induced Expression of SMADs protein. Western blot showed that pirfenidone (0.5 mg/ml) reduced the TGF-β2-induced Expression of SMAD2 and SMAD3 when compared with control cells. The inhibitory effects could be detected but were not statistically significant in the 0.25 mg/ml PFD groups. *P<0.05 versus the corresponding value for control cells. Data are mean ± SD of results from three independent cultures.

In the presence of TGF-β2, PFD reduced the TGF-β2-induced expression of SMAD2 and SMAD3 protein significantly at the concentration of 0.5 mg/ml (*P*<0.05) compared with the control group by western blot analyses. The inhibitory effects could be detected but were not statistically significant in the 0.25 mg/ml PFD groups. (**Figure 10**).

### PFD Inhibited TGF-β2-induced Nuclear Translocation of SMADs

**Figure 11:**
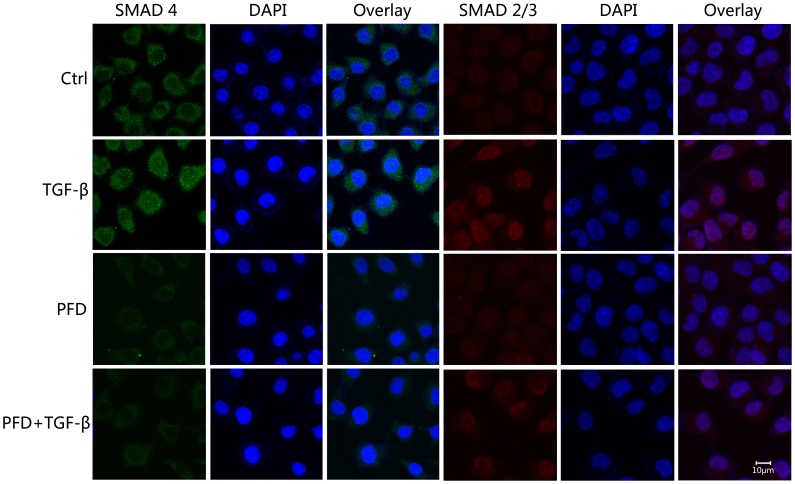
Inhibitory effect of pirfenidone on TGF-β2-induced nuclear translocation of SMADs in SRA01/04 cells under confocal microscopy. After stimulated by 12.5 ng/ml TGF-β2 for 24 hours, the staining of nuclei SMAD4 (green) enhanced when that of SMAD4 in the cytoplasm were weakened, and SMAD2/3 proteins (red) were assembled on the nuclear membrane and some of them entered the nuclei. Expression of nuclei SMAD2/3 and SMAD4 were depressed after treatment with 0.5 mg/ml pirfenidone. The nuclei stained by DAPI (blue). Magnification, ×1000.

In the absence of TGF-β2, staining of nuclei SMAD2/3 and SMAD4 proteins in HLECs were very faint by immunofluorescence assay. After stimulated by 12.5 ng/ml TGF-β2 by 24 hours, nuclear translocation of SMADs could be detected under confocal microscopy: 1.the staining of nuclei SMAD4 enhanced and that of SMAD4 in the cytoplasm were weakened; 2.SMAD2/3 proteins were assembled on the nuclear membrane and some of them entered the nuclei. In the presence of TGF-β2, nuclei SMAD2/3 and nuclei SMAD4 were reduced in the 0.25 and 0.5 mg/ml PFD treated groups compared with the controls under confocal microscopy with magnification of 1000, and the inhibitory effect of 0.5 mg/ml PFD was stronger (**Figure 11**). Therefore, according to our results with immunocytochemistry, PFD could block the TGF-β2-induced nuclear translocation of the Smad2/3 and SMAD4 in HLECs.

## Discussion

This is the first study to show an inhibitory effect of PFD on TGF-β2-induced proliferation, migration and epithlial-mesenchymal transition of HLEC (SRA01/04 cells), the pivotal cell being involved in the development of PCO. The effect seemed to follow a dose-dependent manner. Typan blue exclusion test and LDH assay showed that there was no significant toxic effect at the concentration levels (0–0.5 mg/ml) used in this study. These findings raise the possibility that PFD may represent a new therapeutic agent for the prevention of PCO.

PFD is an anti-inflammatory and anti-fibrotic agent and it exhibits the inhibitory effects on a host of cell types in a range of concentration with 0.2 to 2.0 mg/ml with no or few toxic effect in vitro. PFD significantly inhibits proliferation of hepatic stellate cell (with a maximum effect at 0.19 mg/ml) [23], rat renal fibroblasts (at 0.2 mg/ml) [24], myometrial and leiomyoma cells (at 1.0 mg/ml) [25]. PFD also has an inhibition effect on ocular cells. Our previous study indicated that PFD inhibited proliferation, migration, and collagen contraction of HTFs in vitro at the concentrations range from 0.15 to 0.3 mg/ml with no toxic effect [19], [20]. Kim H et al. reported that PFD (at 1.9 mg/ml) also inhibited the growth of orbital fibroblasts obtained from patients with thyroid-associated ophthalmopathy (TAO) [26]. Choi K et al. proved the inhibitory effect of PFD (from 0.25 to 0.5 mg/ml) on TGF-β-mediated fibrogenesis in the human RPE cell line ARPE-19 [27]. In the current study, we also found that the maximal inhibitory concentration of PFD on the proliferation, migration of HLECs was 0.5 mg/ml and caused no toxic effect. The infinite cell line (SRA01/04) we used was transfected with plasmid vector DNA containing a T antigen of SV40 from primary cultures of HLECs in 1998 [22], which has been widely used in vitro to investigate the underlying mechanisms of cataract formation.

TGF-β2 signaling plays a key role in the development of PCO after cataract surgery. TGF-β2 can activate its down-streaming SMAD pathways and mediate the regulation of trans-differentiation and matrix contraction, and ultimately contribute to the formation of PCO [28]. our group and others studies have previously shown that PFD can down regulate the expression of TGF-βs in HTFs and many other human cells (e.g., HLECs, hepatic stellate cells, and renal fibroblasts) [19], [23], [24], [29]. Choi K et al. have elucidated the TGF-β signaling pathways as the target of the inhibitory effect of PFD and PFD inhibited TGF-β signaling by preventing nuclear accumulation of active Smad2/3 complexes rather than phosphorylation of Smad2/3 in RPE cells [27]. In the current study, we provided new evidence that PFD exhibited its inhibitory effect by depressing TGF-β-SMAD signaling pathway: PFD inhibited protein and gene expression of TGF-β2, depressed the TGF-β2-induced expression of SMADs proteins, and also prevented the nuclear accumulation and translocation of SMADs proteins in HLECs. As we known, beside SMAD2/3/4 nuclear translocation, TGF-β receptor-induced responses are mediated through numerous signaling pathways, such as TGF-β-induced Erk activation and tyrosine phosphorylation, TGF-β-induced JNK/p38 activation and P13K/Akt pathway, to induce a variety of different responses [30]. And there are cross-talks between TGF-β/SMADs and other cell signaling passways [31]. Futher studies are needed to investigated the other molecular medhanisms of PFD for the inhibitory action for TGF-β2-induced fibrogenesis in lens epithelial cells. Taken together, these findings support the concept that PFD is a broad-spectrum anti-fibrotic agent and the effect may be mediated by the TGF-β/SMAD signaling pathway.

In conclusion, PFD shows an inhibiting effect on the proliferation, migration and epithlial-mesenchymal transition of SRA01/04 cells in vitro even in the presence of TGF-β2 stimulation, and the effect follows a dose-response manner. It is unlikely that the inhibition effect is mediated by toxic effect. The inhibition effect may be related to the regulation of TGF-β2/SMAD mRNA and protein expression. We propose that pirfenidone may be a potential antifibrotic agent to prevent the occurrence of PCO in patients after cataract surgery.
